# Supplementary material for: Internal conflict and prejudice-regulation: Emotional ambivalence buffers against defensive responding to implicit bias feedback
Source: PLoS One. 2022 Mar 17;17(3):e0264535. doi: 10.1371/journal.pone.0264535 (PMC8929642; doi:10.1371/journal.pone.0264535)

**Supplemental Materials**

This appendix provides supplemental information for measures and experimental stimuli. Additional information about experimental condition and the exact language for all measures included in and excluded from analyses are available throughout this document.

**Language and Stimuli used for Feedback in Both Sample 1 and 2**

Participants first viewed a consent form and then were randomly assigned to experimental condition. Participants then proceeded to complete a test that they were told would measure “unconscious racial attitudes”. This test was not actually used to provide the feedback, as it was merely a ruse to provide participants with a basis for feedback that was, in reality, randomly manipulated. The test presented them with pictures of men they needed to categorize according to race, with accuracy and speed supposedly being measured for the purpose of yielding a “bias score” that would later be reported to them.

All participants received the following information and graph.

Based on this test, it is clear that you are racially biased at the unconscious level. More specifically, the results indicate that you have a strong preference in favor of White people compared to Black people. Below, you’ll see a graphical representation of where your test scores place you compared to the population.

Please note that a significant amount of research in the social and behavioral sciences indicates that scores on this test predict people’s judgment of and behavior towards racial minorities. For example, one study demonstrated that people with racial bias often avoid being friends with racial minorities or treat them unfairly in social situations, even though they didn’t intend to. This means that there is a really good chance that you harbor unconscious bias towards Black people and are particularly likely to discriminate against them.

Below is a graphical representation of your estimated performance on this test of unconscious racial bias. The figure is not intended to be a perfect representation of your score, but to give you a general sense of how your unconscious racial attitudes compare to other people in the population.


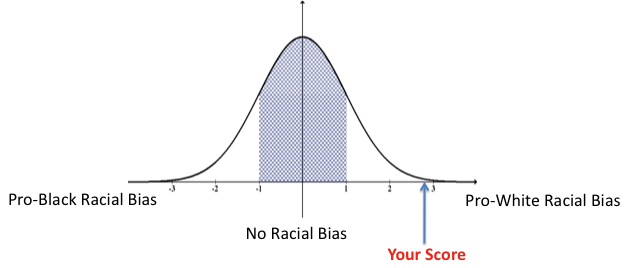


**Measures Included in Both Sample 1 and Sample 2**

**Positive Affect**

*Scale= does not apply at all (1) to applies very much (7).*

1. happy
2. energetic
3. optimistic
4. content
5. good
6. friendly

**Negative Affect**

*Scale= does not apply at all (1) to applies very much (7).*

1. angry at myself
2. guilty
3. regretful
4. annoyed at myself
5. disappointed with myself
6. shame
7. self-critical.

**Defensive Responding**

*Earlier, you completed a test that involved the pairing of photos of Black and White people with positive/negative traits. Psychologists use this as a test of unconscious racial prejudice.*

1. In your opinion, how credible is this test? *(1=Not at all credible, 7= Extremely Credible)*
2. In your opinion, how objective is this test? *(1=Not at all objective, 7= Extremely objective)*
3. In your opinion, how valid are the results of this test? *(1=Not at all valid, 7= Extremely valid)*
4. In your opinion, how useful is this test for understanding people’s racial attitudes? *(1=Not at all useful, 7= Extremely useful)*

**Bias Awareness**

1. How likely is it that your unconscious beliefs are unfavorable toward racial minorities? *(1=Not at all likely, 7=Extremely Likely)*
2. To what extent do you think your unconscious beliefs are unfavorable towards racial minorities? *(1=Not at all, 7= Extremely)*
3. Do you believe that your unconscious racial attitudes affect your judgments towards racial minorities in an unfair way? *(1=Not at all, 7=Yes, definitely)*
4. Do you believe that your unconscious racial attitudes influence your behavior towards racial minorities in an unfair way?  *(1=Not at all, 7=Yes, definitely)*
5. Do you believe that you are unconsciously prejudiced towards racial minorities? *(1=Not at all, 7=Yes, definitely)*
6. How worried are you that you are unconsciously prejudiced towards racial minorities?  *(1=Not at all worried, 7=Extremely worried)*
7. How common is unconscious racial prejudice in America? *(1=Not at all common, 7=extremely common)*
8. How likely is it that unconscious racial attitudes bias people’s judgments and behavior towards racial minorities? *(1=Not at all likely, 7=Extremely Likely)*
9. Do you think differences between racial groups can be explained by the effects of unconscious racial bias and prejudice? *(1=not at all, 7= definitely)*
10. How common is racial prejudice in America? *(1=Not at all common, 7=Extremely common)*
11. How big of a problem is racial prejudice in America? *(1=Not at all a problem, 7=A major problem)*
12. How racially prejudiced is the average American? *(1=Not at all prejudiced, 7= extremely prejudiced)*
13. Do you think that racial minorities are held back in society because of racial prejudice? *(1=Not at all, 7=Yes, definitely)*

**Demographics**

Finally, we would like you to give us a little information about yourself. Before completing this questionnaire, please respond to the following background questions.

What is your age? _________________

Your gender? Male Female

Please indicate your race/ethnicity.

Latino/Hispanic

Black/African American

Asian/Asian American

White/Caucasian

Native American

Other _________________

Are you a U.S. citizen? Yes No

Were you born in the U.S.A? Yes No

What is your total family (including parent income if dependent on parents) income?

______ Less than $10,000

______ $10,000-$19,999

______ $20,000-$29,999

______ $30,000-$39,999

______ $40,000-$49,999

______ $50,000-$59,999

______ $60,000-$69,999

______ $70,000-$79,999

______ $80,000-$89,999

______ $90,000-$99,999

______ $100,000 or greater

What is the highest level of education you have completed?

Grade School

Some High School

High School Diploma or Equivalent

Some College

Associate’s Degree

Bachelor’s Degree

Master’s Degree

Advanced Degree (PhD, DPHIL, J.D., M.D., DDS, etc)

**Excluded Measures from Sample 2**

***Racial Resentment (Kinder & Sanders, 1996)***

1. Over the past few years, blacks have gotten less than they deserve. (Reverse)
2. Irish, Italian, Jewish, and many other minorities overcame prejudice and worked their way up. Blacks should do the same without any special favors
3. It's really a matter of some people not trying hard enough; if blacks would only try harder they could be just as well off as whites.
4. Generations of slavery and discrimination have created conditions that make it difficult for blacks to work their way out of the lower class. (Reverse)

1=agree strongly, 2=agree somewhat, 3=neither agree nor disagree, 4=disagree somewhat, 5=disagree strongly

***Attitudes Toward Blacks Scale (Brigham, 1993)***

1. If a black were put in charge of me, I would not mind taking advice and direction from him or her.
2. If I had a chance to introduce black visitors to my friends and neighbors, I would be pleased to do so.
3. I would rather not have blacks live in the same neighborhood I live in. (Reversed)
4. I would probably feel somewhat self-conscious dancing with a black in a public place.(Reversed)
5. I would not mind it at all if a black family with about the same income and education as me moved in next door.
6. I think that black people living in the United States look more similar to each other than white people do. (Reversed)
7. Interracial marriage should be discouraged to avoid the “who-am-I?” confusion which the children feel. (Reversed)
8. I get very upset when I hear a white make a prejudicial remark about blacks.
9. I favor open housing laws that allow more racial integration of neighborhoods.
10. It would not bother me if my new roommate was black.
11. It is likely that blacks will bring violence to neighborhoods when they move in. (Reversed)
12. I enjoy a funny racial joke, even if some people might find it offensive. (Reversed)
13. The federal governments should take decisive steps to override the injustices blacks suffer at the hands of local authorities.
14. Black and white people are inherently equal.
15. Black people are demanding too much too fast in their push for equal rights in the USA. (Reversed)
16. Whites should support blacks in their struggle against discrimination and segregation in the USA.
17. Generally, blacks are not as smart as whites. (Reversed)
18. I worry that in the next few years I may be denied my application for a job or a promotion because of preferential treatment given to minority group members. (Reversed)
19. Racial integration (of schools, businesses, residences, etc.) has benefitted both whites and blacks.
20. Some blacks in the USA are so touchy about race that it is difficult to get along with them. (Reversed)

Scoring 1 (Strongly Agree) 2 (Agree) 3 (Agree somewhat) 4 (Neutral) 5 (Disagree somewhat) 6 ( Disagree) 7 (Strongly Disagree)

***Social Dominance Orientation (Sidanius & Pratto, 2001)***

INSTRUCTIONS: Using the 7-point scale below, please rate how strongly you agree or disagree with each of the following statements. Circle the number corresponding to your degree of agreement/disagreement with each statement.

Strongly Disagree (1) Disagree (2) Somewhat disagree (3) Neutral or Undecided (4) Somewhat Agree (5) Agree (6) Strongly agree (7)

# 1 2 3 4 5 6 7

_____ 1. Some groups of people are simply inferior to other groups.

_____ 2. In getting what you want, it is sometimes necessary to use force against other groups.

_____ 3. It is OK if some groups have more of a chance in life than others.

_____ 4. To get ahead in life, it is sometimes necessary to step on other groups.

_____ 5. If certain groups stayed in their place, we would have fewer problems.

_____ 6. It is probably a good thing that certain groups are at the top and other groups are at the bottom.

_____ 7. Inferior groups should stay in their place.

_____ 8. Sometimes other groups must be kept in their place.

_____ 9. It would be good if groups could be equal.

_____ 10. Group equality should be our ideal.

_____ 11. All groups should be given an equal chance in life.

_____ 12. We should do what we can to equalize conditions for different groups.

_____ 13. Increased social equality.

_____ 14. We would have fewer problems if we treated people more equally.

_____ 15. We should strive to make incomes as equal as possible.

_____ 16. No group should dominate in society.

***Skepticism about social science (adapted from McCright, Dentzman, Charters, & Dietz (2013)***

1. How much confidence would you say you have in the scientific community? **(***1= no confidence at all, 5= complete confidence)*
2. ‘How much do you distrust or trust social scientists to:’

‘create knowledge that is unbiased and accurate?’

‘create knowledge that is useful?’

‘advise government officials on policy?’

‘inform the public on important issues?

*Scale: 1=completely distrust, 2= partially distrust, 3= neither distrust or trust, 4=partially trust, 5= completely trust*

**Motivation to Control Prejudice Reactions (Dunton & Fazio, 1997)**

*Instructions:* Please indicate your level of agreement with each of the following statements by circling your response on the scale below.

1. In today’s society it is important that one not be perceived as prejudiced in any manner.
2. I always express my thoughts and feelings, regardless of how controversial they might be. (Reversed)
3. I get angry with myself when I have a thought or feeling that might be considered prejudiced.
4. If I were participating in a class discussion and a Black student expressed an opinion with which I disagreed, I would be hesitant to express my own viewpoint.
5. Going through life worrying about whether you might offend someone is just more trouble than it’s worth. (Reversed)
6. It’s important to me that other people not think I’m prejudiced.
7. I feel it’s important to behave according to society’s standards.
8. I’m careful not to offend my friends, but I don’t worry about offending people I don’t know or like. (Reversed)
9. I think that it is important to speak one’s mind rather than to worry about offending someone (Reversed)
10. It’s never acceptable to express one’s prejudices.
11. I feel guilty when I have a negative though or feeling about a Black person.
12. When speaking to a Black person, it’s important to me that he/she not think I’m prejudiced.

**System justification (Kay & Jost, 2003)**

1. In general, you find society to be fair.
2. In general, the American political system operates as it should.
3. American society needs to be radically restructured.
4. The United States is the best country in the world to live in.
5. Most policies serve the greater good.
6. Everyone has a fair shot at wealth and happiness.
7. Our society is getting worse every year.
8. Society is set up so that people usually get what they deserve.

Scale: 1=Strongly Disagree, 9 = Strongly agree

**Perception of Social Scientists and Research**

1. Social scientists think that all White people are racists. *(1=Not at all, 7= Yes, definitely)*
2. Social scientists think that all Black people are treated unfairly because of their race. *(1=Not at all, 7= Yes, definitely)*
3. Social scientists think that the only reason Black people don’t succeed as much as White people is because they are victims of racial prejudice. *(1=Not at all, 7= Yes, definitely)*
4. How infected by political motives and values are social scientists? *(1=Not at all, 7= Very much so)*
5. To what extent are social scientists who study the psychology of unconscious racial bias motivated by a political or ideological agenda? *(1=Not at all, 7= Yes, definitely)*
6. How credible are social scientists who study the psychology of unconscious racial bias? *(1=Not at all credible, 7=extremely credible)*
7. How objective are social scientists who study the psychology of unconscious racial bias? *(1=Not at all objective, 7=extremely objective)*
8. Social scientists specialize in lying with numbers. *(1=Strongly Disagree, 7=Strongly Agree)*

**Attitudes towards interventions to reduce implicit bias in organizational contexts, law enforcement, and society generally.**

1. In your opinion, how important is it that the law requires employers to undergo training to reduce their unconscious racial bias? *(1=Not at all important, 7=Extremely important)*
2. In your opinion, how important is it that organizations require their employees to undergo training to reduce their unconscious racial bias? *(1=Not at all important, 7=Extremely important)*
3. In your opinion, how important is it that law enforcement personnel undergo training to reduce their unconscious racial bias? *(1=Not at all important, 7=Extremely important)*
4. In your opinion, how valuable are anti-bias programs in eliminating racial discrimination in the workplace? (1=Not at all valuable, 7=Extremely valuable)
5. In your opinion, how valuable are anti-bias programs in eliminating racial discrimination in the criminal justice system? (1=Not at all valuable, 7=Extremely valuable)
6. In your opinion, how valuable are anti-bias programs in eliminating racial discrimination in the society? (1=Not at all valuable, 7=Extremely valuable)

***Authoritarianism (Stenner, 2005)***

Please read each pair of qualities that children might have, and indicate which of the two is the most desirable quality for a child to have.

Independence or Respect for Elders: 1. Independence is more desirable. 2. Respect for Elders is more desirable 3. I’m not sure 4. Both are equally important 5. Neither is very important

Obedience or Self-reliance 1. Obedience is more desirable 2. Self-reliance is more desirable 3. I’m not sure 4. Both are equally important 5. Neither is very important

Curiosity or Good Manners: 1. Curiosity is more desirable 2. Good Manners are more desirable 3. I’m not sure 4. Both are equally important 5. Neither is very important

Being Considerate or Well-behaved: 1. Being Considerate is more desirable 2. Being Well-behaved is more desirable 3. I’m not sure 4. Both are equally important 5. Neither is very important

***Internal/External Motivation to Control Prejudice (Plant & Devine, 1998)***

*
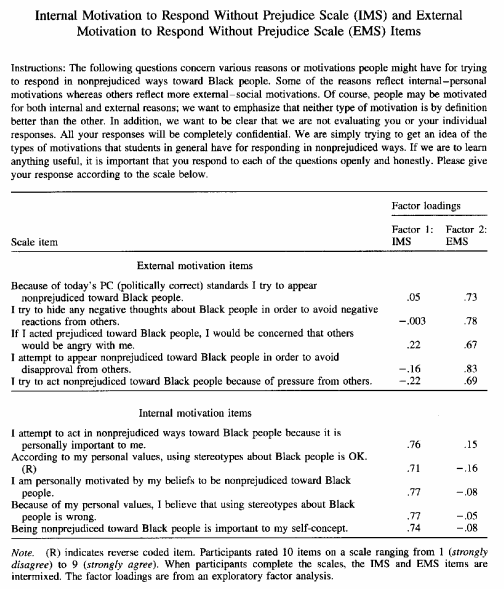
*

**Additional Experimental Condition in Sample 1**

In study 1, an additional experimental condition was run concomitantly with the other conditions described in the main paper, but for reasons not relevant to the hypotheses we test in the current paper. This condition involved providing participants with egalitarian feedback. Specifically, after completing the Race IAT, participants in the egalitarian feedback condition received the following information and graph:

Based on this test, it is clear that you are racially fair at the unconscious level. More specifically, the results indicate that you have an equal preference for White people and Black people.

Please note that a significant amount of research in the psychological sciences indicates that scores on this test predict people’s judgment of and behavior towards racial minorities. For example, one study demonstrated that White employers who scored lower on measures of unconscious bias evaluated White job applicants and Black job applicants fairly. This means that there is a really good chance that you do NOT harbor unconscious bias towards Black people and are particularly likely to treat people of different races fairly.

Below is a graphical representation of your estimated performance on this test of unconscious racial bias. The figure is not intended to be a perfect representation of your score, but to give you a general sense of how your unconscious racial attitudes compare to other people in the population.


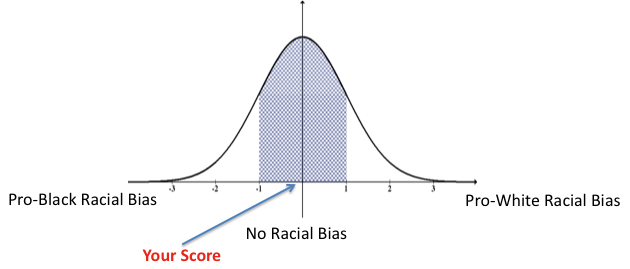

Supplement: S1 File — (DOCX) [file pone.0264535.s001.docx]
