# Supplementary material for: Internal conflict and prejudice-regulation: Emotional ambivalence buffers against defensive responding to implicit bias feedback
Source: PLoS One. 2022 Mar 17;17(3):e0264535. doi: 10.1371/journal.pone.0264535 (PMC8929642; doi:10.1371/journal.pone.0264535)
Supplement: Attachment — Submitted filename: Response to Reviewers.docx [file pone.0264535.s003.docx]

January 19, 2022

Dear Dr. Shook,

Thank you for inviting us to revise and resubmit the manuscript (PONE-D-21-23604) entitled Internal Conflict and Prejudice-Regulation: Emotional Ambivalence Buffers Against Defensive Responding to Implicit Bias Feedback for publication consideration in *PLOS ONE*. We have addressed each comment in the revised manuscript and have responded to each point raised by yourself and the reviewer(s) below.

**Comments from the Editor**

***Thank you for submitting your manuscript to PLOS ONE. After careful consideration, we feel that it has merit but does not fully meet PLOS ONE’s publication criteria as it currently stands. Therefore, we invite you to submit a revised version of the manuscript that addresses the points raised during the review process.***

***I greatly appreciate your responsiveness to the first round of reviews. You have done a very thorough job of addressing the reviewers' and my initial concerns, and the manuscript is much stronger.***

**Our Response:** Thank you so much.

***However, a point of clarification has come up with the revised manuscript, regarding the proposition that emotional ambivalence in the current studies is incidental. As the assessment of positive and negative emotion occurred right after receiving bias feedback (or not) and the IAT, it is hard to believe that participants' current emotions were unrelated to study procedures. Ideally, incidental emotions would have been assessed prior to the manipulation. Although the emotional ambivalence index did not differ by condition, did positive or negative emotion differ by condition? A stronger case for emotions being incidental in these studies needs to be made, and the concern about the procedure should be noted in the limitations.***

**Our Response:** We appreciate this question. In the last round, we conceptualized emotional ambivalence as a state experience of the simultaneous positive and negative emotions “in the present moment” and triggered by prior, unrelated experiences. We built on prior scholarship Rees et al., (2013) where *incidental* emotional ambivalence was manipulated. We did this because although we measured emotional ambivalence following bias feedback (or not) from the IAT, we conceptualized these as independent constructs. We were not studying emotional ambivalence “about their biases” or “about the feedback” directly. Rather, we were studying a more general affective state of emotional ambivalence “in the present moment” in order to unpack a more general effect of experiencing positive and negative emotions simultaneously (irrespective of the target). That is, the feedback does not cause the ambivalence in our model, and the ambivalence is not about their IAT performance; their ambivalence as an independent state is proposed to make them less defensive to the feedback. We can see how this might be confusing since it is measured after the feedback, and could lead to the erroneous conclusion that we did this because we were proposing a causal link. We are not. Being in a state of ambivalence does not require negative feedback and is not a reaction to it.

Supporting our conceptualization of emotional ambivalence as being an independent construct from feedback, we found no statistical relation between the feedback condition and participants’ reported experiences of emotional ambivalence. This is not to say that feedback does not impact people emotionally. It just did not impact, nor was it introduced in order to impact, ambivalence. The feedbacks’ emotional impact was exerted on univalent affect. There was a significant positive relationship between feedback condition and the experience of negative emotions “in the present moment” and a significant negative relationship between feedback condition and the experience of positive emotions “in the present moment”.

As a result of diverging effects of bias feedback on univalent affect, emotional ambivalence, as we predicted, was not observed in response to the bias feedback. This independence of feedback and emotional ambivalence is important because we want to emphasize that feedback is threatening and an emotional state that is independent from the feedback (emotional ambivalence) can reduce the threat and the defensiveness to that feedback. We find support for both predictions: emotional ambivalence is not only independent of the feedback but can attenuate such defensiveness, even more than negative emotions. We have revised our abstract and introduction to make this clearer.

That being said, and based on your comments, we have played down the discussion of “incidental” emotions in our revised manuscript. Indeed, although feedback was statistically unrelated to the experience of state emotional ambivalence (p > .3), you are correct that we cannot be entirely sure that all participants reported emotional ambivalence about an unrelated experience. It is possible that some of them may have reported experiencing emotional ambivalence “in the present moment” while thinking about their feedback on the IAT they had just received. For this reason, we now raise the issue about the timing of the emotional ambivalence measure (after feedback and IAT) in the limitations section, suggesting exciting opportunities for future research.

***Review Comments to the Author

Please use the space provided to explain your answers to the questions above. You may also include additional comments for the author, including concerns about dual publication, research ethics, or publication ethics. (Please upload your review as an attachment if it exceeds 20,000 characters)***

***Reviewer #1: I was perhaps a bit too dismissive of the ambivalence angle and instead fixating on mere negative affect in my first round review. I appreciate that the authors took my confusion as an opportunity to clarify their logic, especially regarding incidental vs. integral affect.***

**Our Response:** We appreciated the opportunity and we thank you for your probing questions.

***However, now I see a clearer disconnect between the logic laid out in the intro and the methods. There’s nothing “incidental” about the emotions people experience immediately after IAT bias feedback, so perhaps I’m missing something when the authors state, “the feelings are not about the feedback.” Why provide the feedback then? I still struggle with the logic here. Did the bias feedback not affect univalent affect? Is emotional ambivalence not computed as a function of positive and negative univalent affect?***

***It’s really a confusing causal argument, especially given the order of events. Participants take an IAT, get bias feedback (or not), and then complete measures of affect, defensiveness, and bias awareness. The authors are claiming a (moderated) causal relationship between bias feedback and defensiveness, but not a causal a relationship between bias feedback and affect? And then claim that that whatever affect participants experience is “incidental” even though the measure of affect followed the bias feedback? It’s just hard to wrap my head around.

It’s also important to clarify that positive and negative affect alone aren’t driving the defensive responding or bias awareness, and I appreciate the authors’ new analyses that clarify that ambivalence is doing work above and beyond univalent affect.

That said, I still struggle with what those analyses are doing; they are not straightforward and do not give a full view of the pattern of data. The most defensive people, according to Figure 1, are those who received bias feedback and who were the least ambivalent. And it doesn’t really look like the ambivalent people are more open. It just looks like they’re no more defensive than people who weren’t provided IAT feedback. So can it be said that emotional ambivalence opens people up and makes them less defensive?***

***My summary assessment is that the logic is a bit strange, the design still puzzles me, and the analyses don’t appear to provide a full, thorough window into what the data actually show. I’ve read and re-read it several times and still have a hard time wrapping my head around it.***

**Our Response:** You have a series of points here that we respond to in turn. First, we provide feedback to participants about their bias in order to create an ecologically valid context in which individuals experience a self-threatening form of feedback that has been previously shown to induce defensiveness (Howell et al., 2016; Vitriol & Moskowitz, 2021). We are interested in whether emotional ambivalence is a moderator of the effect of feedback on increased defensiveness. We predicted emotional ambivalence would attenuate this relationship because it increases cognitive flexibility.

Second, although we measured emotional ambivalence following receiving bias feedback (or not) from the IAT, we conceptualized these as independent constructs. We were not studying emotional ambivalence “about their biases” or “about the feedback” directly. Rather, we were studying a more general affective state of emotional ambivalence “in the present moment” in order to unpack a more general effect of experiencing positive and negative emotions simultaneously (irrespective of the target). Supporting our conceptualization of emotional ambivalence as being an independent construct from feedback, we found no statistical relation between the feedback condition and participants’ reported experiences of emotional ambivalence. In contrast, there was a significant positive relationship between feedback condition and the experience of negative emotions “in the present moment” and a significant negative relationship between feedback condition and the experience of positive emotions “in the present moment”. As a result, we feel confident that emotional ambivalence – reporting that they experienced high levels of both positive and negative emotions at the same time in the present moment - was not a response to the bias feedback. The independence of feedback and emotional ambivalence is important because we conceptualize emotional ambivalence as a moderator. Prior research has shown that feedback is threatening (Howell et al., 2015; Vitriol & Moskowitz, 2021) and we suggest that an emotional state that is independent from the feedback can reduce threat and defensiveness. We find support for both predictions: emotional ambivalence is not only independent of the feedback but can attenuate such defensiveness, even more than negative emotions. We have revised our abstract and introduction to make this clearer.

Third, based on your comments, we have played down the discussion of “incidental” emotions in our revised manuscript. Thank you for challenging us to think deeper about this. Indeed, although feedback was statistically unrelated to the experience of state emotional ambivalence, you are correct that we cannot be entirely sure that all participants reported emotional ambivalence about an unrelated experience. It is possible that some of them may have reported experiencing emotional ambivalence “in the present moment” while thinking about their feedback on the IAT that they had just received. Because we cannot be absolutely certain that the emotional ambivalence was incidental to the current situation, we have removed this discussion. We have also raised the issue about the timing of the emotional ambivalence measure (after feedback and IAT) in the limitations section, suggesting exciting opportunities for future research.

Fourth, we have also removed mention that emotional ambivalence “opens people up” as it is more accurate to say that it “reduces defensiveness” which we empirically measure directly.

Thank you for the prompts to be clearer with our writing.
